# Bandwidth of gamma-distribution-shaped functions via Lambert W function

**DOI:** 10.1016/j.spl.2026.110707

**Published:** 2026-03-06

**Authors:** Anthony LoPrete, Johannes Burge

**Affiliations:** a Bioengineering Graduate Group, University of Pennsylvania, Philadelphia, PA, USA; b Neuroscience Graduate Group, University of Pennsylvania, Philadelphia, PA, USA; c Department of Psychology, University of Pennsylvania, Philadelphia, PA, USA

**Keywords:** Probability theory, Full-width at half maximum, Special functions, primary 60E05, secondary 33E20

## Abstract

The full width at half maximum (FWHM) is a useful quantity for characterizing the bandwidth of unimodal functions. However, a closed-form expression for the FWHM of gamma-shaped functions – i.e. functions that are shaped like the gamma distribution probability density function (PDF) – is not widely available. Here, we derive and present just such an expression. To do so, we use the Lambert W function to compute the inverse of the gamma PDF. We use this inverse to derive an exact analytic expression for the full-width at an arbitrary *y*-proportion of the maximum (FWyM) of a gamma distribution, from which the FWHM follows trivially. (An expression for the octave bandwidth of gamma-shaped functions is also provided.) The FWHM is then compared to the Gaussian approximation of gamma-shaped functions. A MATLAB function is provided that computes the key quantities. A few other related issues are also discussed.

## 1. Introduction

Functions that are shaped like gamma distribution probability density functions (PDFs) are widely used across science, engineering, and business. In vision research and systems neuroscience, gamma-shaped functions are used to characterize the temporal response properties of photoreceptors to photon absorptions (Cao et al., 2007), the distribution of contrast energy across images of natural scenes (Wainwright and Simoncelli, 1999; Iyer and Burge, 2019), and the characterization of cross-correlograms obtained from continuous psychophysics experiments (Chin and Burge, 2022; Burge and Bonnen, 2025; Burge and Cormack, 2024; Bonnen et al., 2015). In archaeology and other fields that make use of radioisotope decay, gamma distributions are useful for radiocarbon dating (Blaauw and Christen, 2011). In electrical engineering and digital signal processing, gamma distributions are often used to model impulse response functions, signal detection, and/or signal-to-noise ratios (Atapattu et al., 2011; Guo et al., 2014). In operations research, gamma distributions are used in queuing theory to describe the distribution of wait times (Allen, 1978). In Bayesian statistics, the gamma distribution is the conjugate prior for Poisson likelihood functions (Gelman et al., 1995). Its widespread use is partly due to the fact that the gamma distribution is a super-family distribution that contains the exponential, chi-squared, and Erlang distributions as special cases.

Gamma-distribution-shaped functions have the shape of gamma distribution PDFs but do not necessarily integrate to 1.0 and can have their domains shifted. Specifically, gamma-distribution-shaped functions are given by

(1) $$g\left(x^{\prime }\right)=Kx^{\prime a-1}\exp\left(-x^{\prime }/b\right)+C$$

where exp(·) denotes the exponential function, $x^{\prime }=x+s$ where *s* is an arbitrary shift to the domain, *a* > 0 is the shape parameter, *b* > 0 is the scale parameter, *K* is an arbitrary scalar determining peak height, and. *C* is an arbitrary constant determining offset from baseline. Gamma-distribution-shaped functions can be converted to gamma distribution PDFs by setting *s* = 0, *C* = 0, and *K* to a scalar that normalizes the area under the curve to 1.0 (see Eq. (3)).

**Fig. 1.**
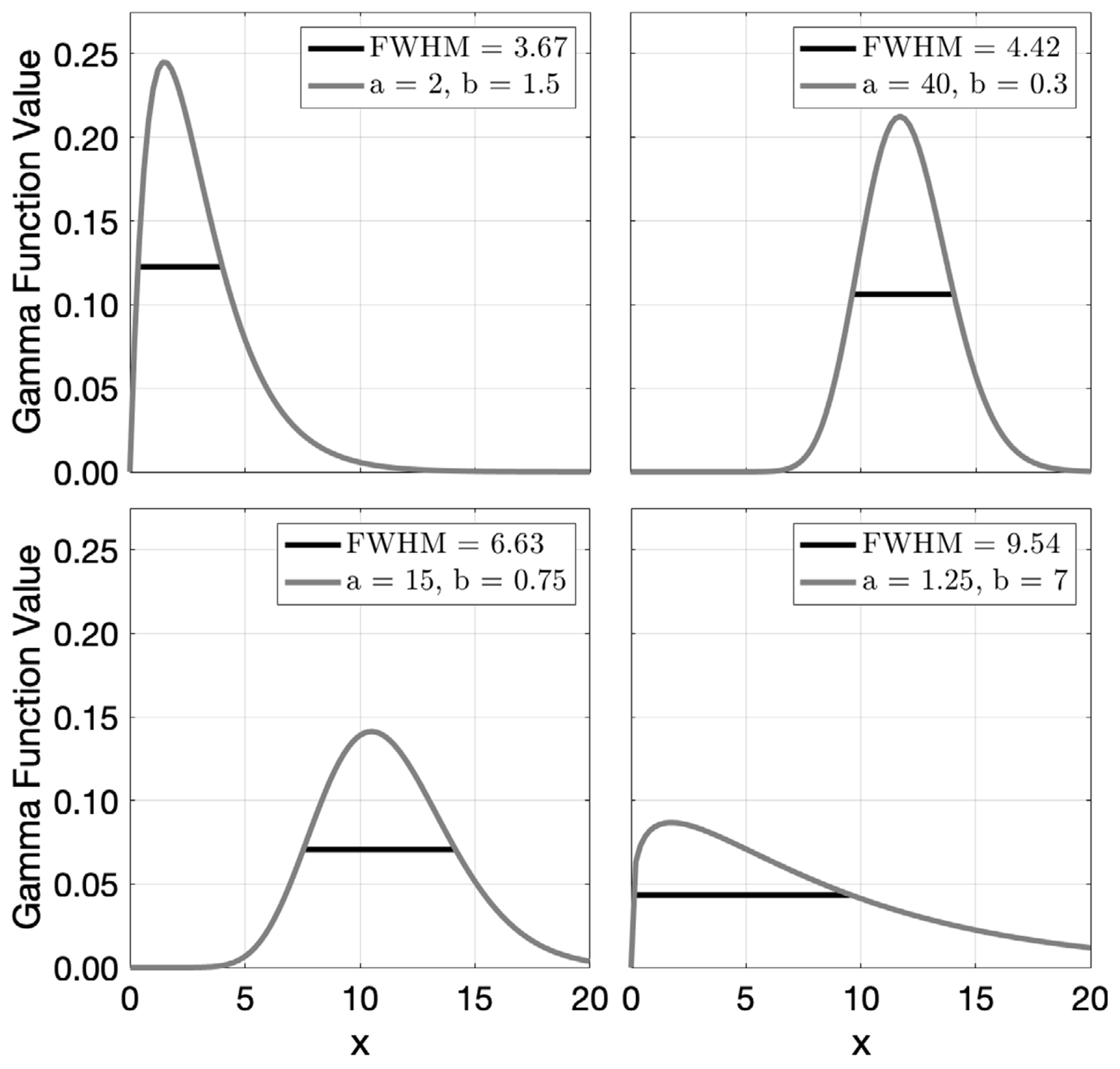
Example gamma probability densities (i.e. gamma-shaped functions; gray curves) and their respective FWHMs (black lines). Values for the FWHM, and the shape and scale parameters are indicated in the legend.

Scientists and engineers often find it useful to describe a function by its spread. The full width at half maximum (FWHM) is a common such descriptor. The FWHM is defined as the distance between arguments corresponding to half the function’s maximum (Fig. 1).^1^ To find the exact value for the FWHM of a unimodal function, one can find an expression for the function inverse, evaluate it at half the function’s maximum value, and then subtract the lesser from the greater output. However, an exact closed-form expression for the FWHM of gamma-distribution-shaped functions is not readily available in the literature.

Here, we derive a closed-form expression for the full width at an arbitrary proportion of the maximum (FWyM) of gamma-distribution-shaped functions, from which an expression for the FWHM trivially follows.

For gamma-distribution-shaped functions, the FWHM – and, more generally, the FWyM – is defined only when the shape parameter *a* is greater than or equal to one. When the shape parameter *a* is less than one, the gamma distribution does not have a properly defined mode; the consequence is that the half-maximum – or any other proportion of the maximum – is not well-defined. For shape parameter *a* = 1, the gamma distribution corresponds to the exponential distribution, for which the FWHM is widely known and can be easily determined via algebraic methods (i.e. FWHM_exp_ = *b* ln 2). To our knowledge, FWHMs have not been published for the other well-established special cases (e.g., Erlang distribution, chi-squared distribution) or, more generally, for the gamma distribution with shape parameter *a* > 1. This article, therefore, focuses on gamma-distribution-shaped functions satisfying this property.

**Fig. 2.**
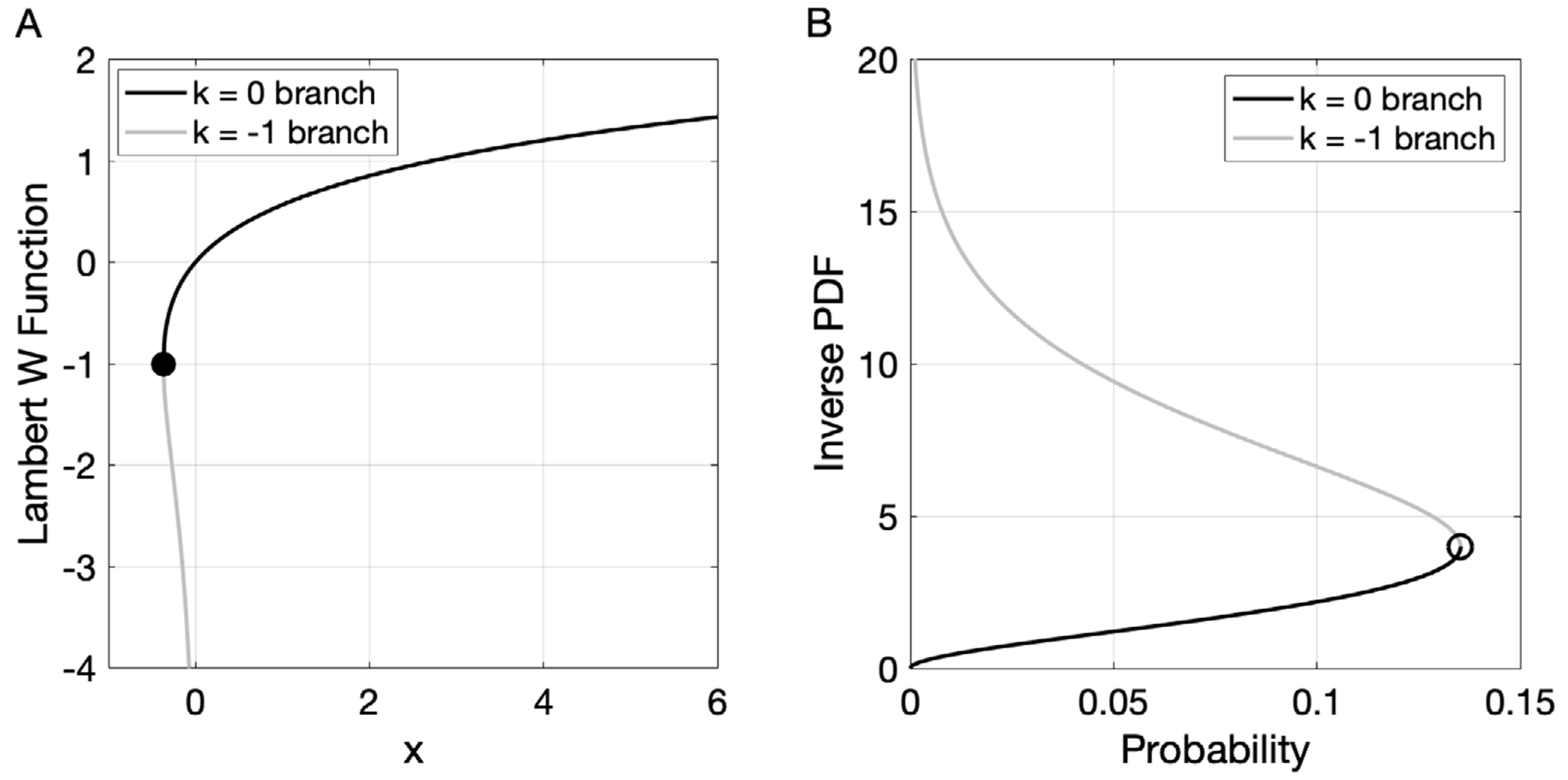
**A** Real-valued branches of the Lambert W function for the *k* = 0 branch (black) and *k* = −1 branch (gray). Both branches equal −1 when evaluated at *x* = −1/*e* (black dot). **B** The value of the inverse of the Gamma(3,2) probability density evaluated using the: *k* = 0 and *k* = −1 branches of the Lambert W function (black and gray, respectively). Note that the *k* = −1 and *k* = 0 branches respectively return values above and below the mode (white dot) of the gamma density.

The main result of the paper is as follows: the FWHM for all gamma-distribution-shaped functions with shape parameter *a* > 1 and scale parameter *b* is given by

(2) $$\mathrm{FWHM}=(a-1)b\left[W_{0}\left(-\frac{2^{-1/(a-1)}}{e}\right)-W_{-1}\left(-\frac{2^{-1/(a-1)}}{e}\right)\right]$$

where *W_k_* is the *k*th branch of the Lambert W function (i.e. $\left.W_{k}(\cdot ),k\in \{0,-1\}\right)$.^2^ The Lambert W function inverts expressions of the form *u* exp (*u*), such that *W_k_* (*u*exp(*u*)) = *u* (Fig. 2). Such an expression appears when deriving the FWHM for gamma-distribution-shaped functions (see Results). The Lambert W function thus makes finding the FWHM of a gamma-shaped function straightforward (Veberič, 2012). However, the Lambert W function is not as well-known as it perhaps ought to be (Kesisoglou et al., 2021), which may be why an analytical expression of the gamma FWHM is not readily available in the literature. Implementations of the Lambert W function are available in many high-level scientific programming languages and computer algebra systems (e.g. MATLAB’s Symbolic Math Toolbox, Python’s SciPy package, Mathematica, Maple, Julia, etc.) with robust real-branch selection on [−1/*e*, 0). Hence, it is simple to compute the FWHM of gamma-distribution-shaped functions using modern scientific computing packages (see 2.5).

We derive the gamma distribution’s inverse PDF – or more precisely, the converse relation – and use it to write an expression of the FWyM from which the FWHM follows trivially. We compare this analytic FWHM to a Gaussian approximation of the gamma distribution’s FWHM. We also present an analytic expression for the octave bandwidth of gamma-distribution-shaped functions. Lastly, we briefly discuss a MATLAB function that implements the main results of this article.

## 2. Results

This section presents the mathematical results of the paper. The preliminary details – the definition of gamma-distribution-shaped functions, the definition of the FWHM, and the Lambert W function – are presented in the introduction. Our derivations use the gamma distribution PDF because it is the most common gamma-distribution-shaped function (see Eq. (3)), and because arbitrary scalings and shifts to the gamma distribution PDF do not change the results.

If a random variable is gamma distributed – that is, *X* ~ Gamma(*a, b*) – with support: *x* ≥ 0, then its probability density *p*(*x*) is given by

(3) $$p(x)=\left(\frac{1}{\Gamma (a)b^{a}}\right)x^{a-1}\exp(-x/b)$$

where *Γ*(·) is the gamma function. The gamma function is a generalization of the factorial function $\Gamma (x)=\left(x-1\right)$! such that for non-integer values of x, the gamma function smoothly interpolates between the outputs of the integer-valued factorial function.

### 2.1. Gamma probability density and its inverse

Here, we derive the inverse of the gamma probability density function. For notational simplicity, we denote probability *p*(*x*) as *p*. Multiplying both sides of Eq. (3) by *Γ*(*a*)*b^a^* and raising both sides to power 1/(*a* – 1) yields

(4) $${\left(p\Gamma \left(a\right)b^{a}\right)}^{\frac{1}{a-1}}=x\exp\left(\frac{-x}{\left(a-1\right)b}\right)$$

Multiplying both sides by −1(*a*−1)*b*) gives

(5) $$\frac{-{\left(p\Gamma (a)b^{a}\right)}^{1/(a-1)}}{(a-1)b}=\frac{-x}{(a-1)b}\exp\left(\frac{-x}{(a-1)b}\right)$$

Substituting *u* = −*x*/((*a*−1)*b*) simplifies the right-hand side

(6) $$\frac{-{\left(p\Gamma \left(a\right)b^{a}\right)}^{1/(a-1)}}{\left(a-1\right)b}=u\exp\left(u\right)$$

Applying the Lambert W function to both sides

(7) $$W_{k}\left(\frac{-{\left(p\Gamma (a)b^{a}\right)}^{1/(a-1)}}{(a-1)b}\right)=W_{k}(u\exp(u))$$

Simplifying the right-hand sight using *u* = *W_k_* (*u* exp(*u*)) and resubstituting −*x*/((*a*−1)*b*) = *u* gives

(8) $$W_{k}\left(\frac{-{\left(p\Gamma (a)b^{a}\right)}^{1/(a-1)}}{(a-1)b}\right)=\frac{-x}{(a-1)b}$$

Finally, multiplying through by *b*(*a*−1) and substituting $f_{k}^{-1}(p)$ for *x* gives

(9) $$f_{k}^{-1}(p)=-(a-1)bW_{k}\left(\frac{-{\left(p\Gamma (a)b^{a}\right)}^{1/(a-1)}}{(a-1)b}\right)$$

Hence, Eq. (9) is the inverse of the gamma probability density function.

Recall that our aim is to obtain the arguments on each side of the mode of the density function that correspond to the desired proportion of the function maximum. For this purpose, the relevant branches of the Lambert W function are indexed at *k* = 0 and *k* = −1 (Fig. 2A). Evaluating Eq. (9) for the *k* = 0 branch of the Lambert W function returns the argument below the mode, while evaluating at the *k* = −1 branch returns the argument above the mode (Fig. 2B). When evaluated at the mode itself, the output is equal for both branches.^3^

### 2.2. Full width of functions (FWyM and FWHM)

The full width at y-max (FWyM) of a unimodal function is the full width of the function at an arbitrary proportion of the maximum (Salvi et al., 2023; Vestergaard et al., 2000). The FWHM is the distance between half-maxima on each side of the mode. To find the FWyM, we evaluate the inverse of the gamma probability density function (Eq. (9)) at the *k* = −1 and *k* = 0 branches with a value that is the desired proportion of the function maximum, and then subtract the smaller from the larger value.

The mode of the gamma probability density is given by (*a*−1)*b*. The desired proportion *y* of the maximum: *p*((*a*−1)*b*) is given by

(10) $$y[p((a-1)b)]=\frac{y}{\Gamma (a)b^{a}}((a-1)b{)}^{a-1}e^{-((a-1)b)/b}$$

Substituting Eq. (10) into Eq. (9) yields

(11) $$x=-(a-1)bW_{k}\left(\frac{1}{(a-1)b}{\left(-\frac{y}{\Gamma (a)b^{a}}((a-1)b{)}^{a-1}e^{-((a-1)b)/b}\Gamma (a)b^{a}\right)}^{1/(a-1)}\right)$$

Simplifying

(12) $$x=-(a-1)bW_{k}\left(-\frac{y^{-1/(a-1)}}{e}\right)$$

The FWyM is the difference between the two values of: *x* obtained from this equation corresponding to the two relevant branches of the Lambert W function. Note that for $0<y<1,z=-y^{1/(a-1)}/e\in [-1/e,0)$, so both *W*_0_(*z*) and *W*_−1_(*z*) are real. The *k* = −1 branch returns the (larger) value above the mode, and the *k* = 0 branch returns the (lesser) value below the mode. Hence, the FWyM is given by

(13) $$\mathrm{FWyM}=\left(-(a-1)bW_{-1}\left(-\frac{y^{-1/(a-1)}}{e}\right)\right)-\left(-(a-1)bW_{0}\left(-\frac{y^{-1/(a-1)}}{e}\right)\right)$$

**Fig. 3.**
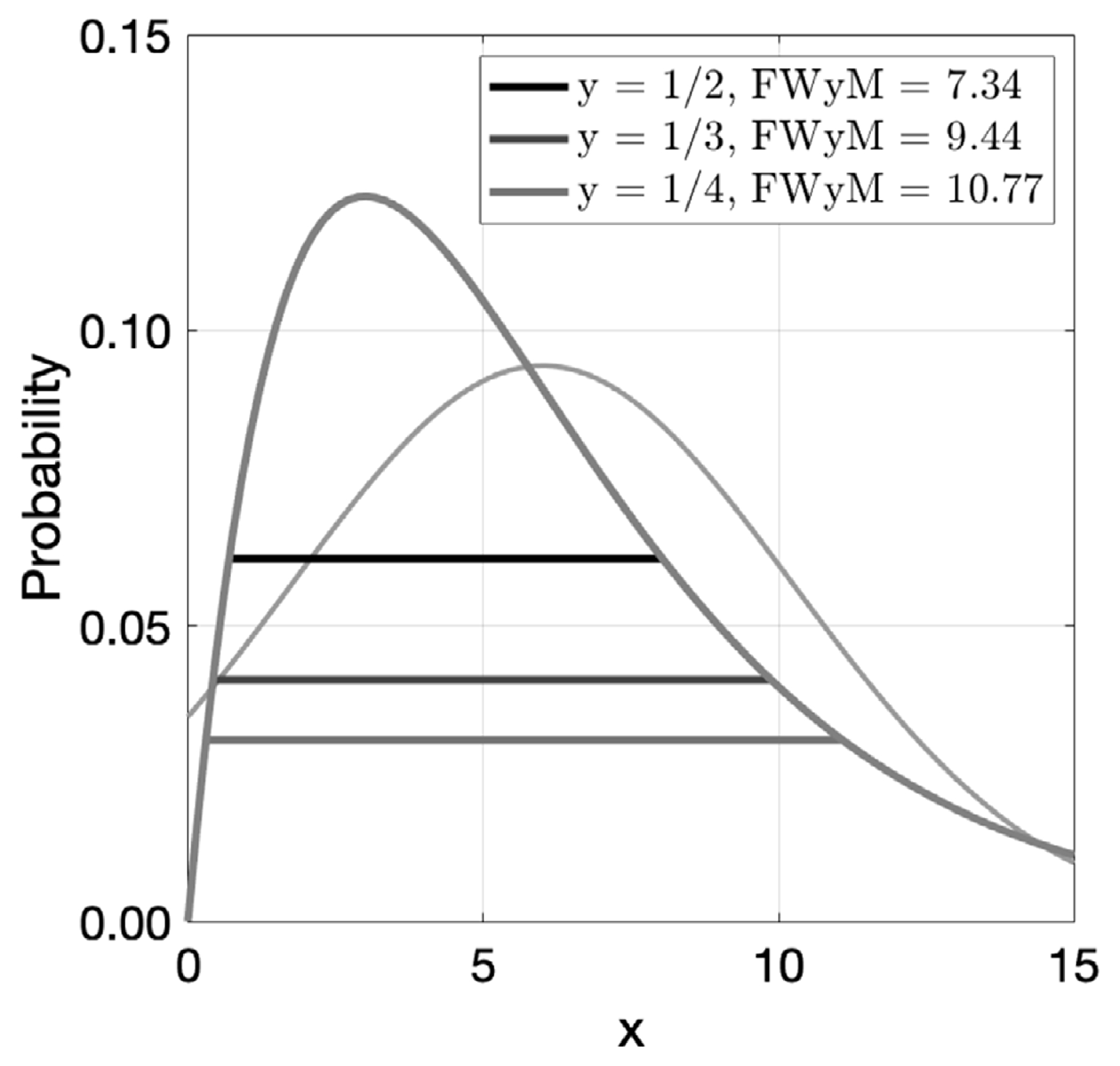
FWyMs of the Gamma(2,3) distribution for three different values of*y* (i.e. 1/2, 1/3, 1/4).

Simplifying and re-arranging yields

(14) $$\mathrm{FWyM}=(a-1)b\left[W_{0}\left(-\frac{y^{-1/(a-1)}}{e}\right)-W_{-1}\left(-\frac{y^{-1/(a-1)}}{e}\right)\right]$$

where *y* can be set to any positive value between 0 and 1 to obtain the full width of the gamma density at any arbitrary proportion of the maximum (see Fig. 3). This expression for the FWyM of the gamma probability density holds for all gamma-distribution-shaped functions for which the FWyM is well defined (i.e. shape parameter *a* > 1). As noted, the most widely used version of this expression is the full width at half maximum (FWHM), which is obtained by simply setting *y* = 1/2 (see Eq. (2)).

### 2.3. Comparison to Gaussian approximation

The gamma distribution is well-approximated as a Gaussian distribution for large values of the shape parameter *a*. The central limit theorem holds that the sum of *n* independently identically distributed (i.i.d.) random variables *X*_1_, *X*_2_, …,*X*_n_ tends toward Gaussian

(15) $$\sum_{i=1}^{n}X_{i}\dot{∼}𝒩\left(n\mu ,n\sigma^{2}\right)$$

where each random variable *X_i_* has the same mean *μ* and variance *σ*^2^. The Gamma(*a, b*) distribution can be described as the sum of *a* i.i.d exponentially distributed random variables with mean *b*

(16) $$\mathrm{Gamma}(a,b)∼\sum_{i=1}^{a}X_{i}\;\text{where}\;X_{i}∼\exp(b)$$

The mean (i.e. expected value) and variance of each random variable *X_i_* equal E[*X_i_*] = *μ_i_* = *b* and $\mathrm{Var}\left[X_{i}\right]=\sigma_{i}^{2}=b^{2}$, respectively. Hence, from the central limit theorem, the random variable *X* ∼ Gamma(*a, b*) is approximately distributed as

(17) $$X\dot{∼}𝒩\left(\mathrm{ab},{\mathrm{ab}}^{2}\right)$$

where mean E[*X*] = *μ* = *ab* and the variance $\mathrm{Var}[X]=\sigma^{2}=ab^{2}$.

The FWHM of a Gaussian distribution is given by $2\sqrt{2log2}\sigma$. Substituting $\sigma =b\sqrt{a}$ yields the relevant Gaussian approximation

(18) $${\mathrm{FWHM}}_{\text{gauss}}=(2\sqrt{2\log2})b\sqrt{a}$$

For large values of *a*, the approximation approaches the true value of the gamma-distribution FWHM. However, the Gaussian-approximate value always overestimates the true value.

**Fig. 4.**
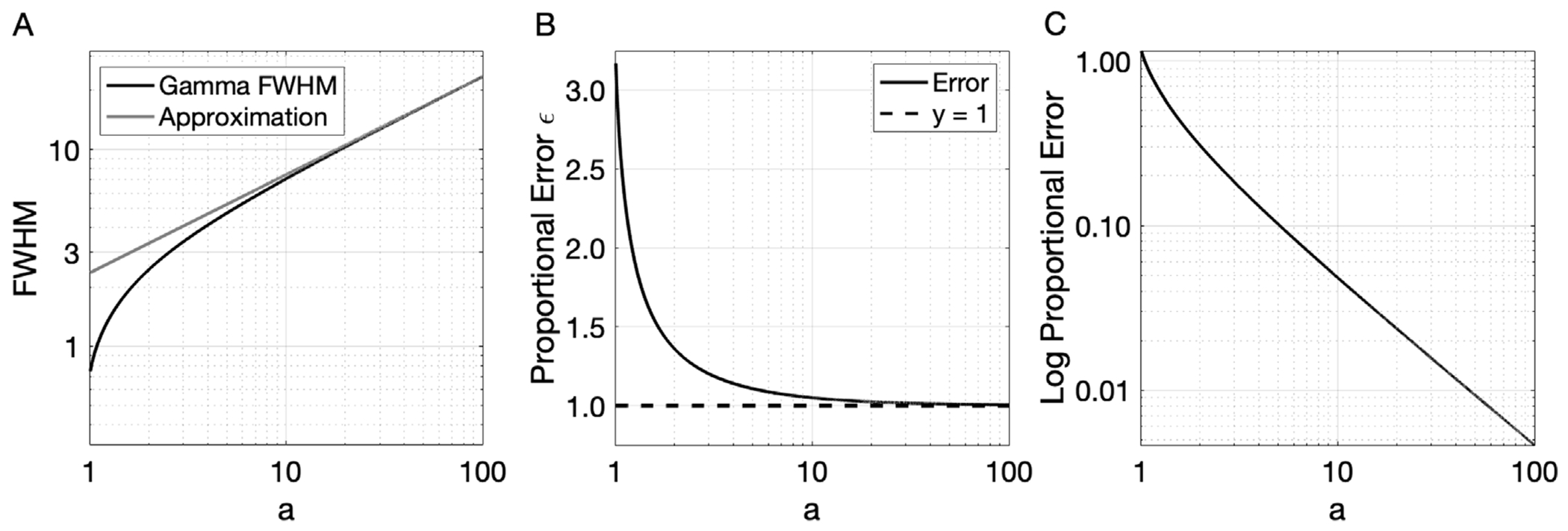
Gamma FWHM versus its Gaussian-approximation. **A** FWHM as a function of the shape parameter *a*, for gamma distributions with scale parameter *b* = 1. **B** Proportional error between the Gaussian-approximation and true value of the FWHM. Note that the proportional error function is invariant to the value of the scale parameter b. **C** Logarithm of the proportional error between Gaussian-approximate and analytical gamma FWHM plotted against *a* on a log-x log-y plot.

Fig. 4 examines the relationship between the Gaussian-approximate and true values of the FWHM for gamma-distribution-shaped functions. It shows the proportion by which the approximation exceeds the true FWHM, the proportional overestimation error (i.e. the ratio between the approximate and true values), and the log proportional error. The proportional overestimation error asymptotically approaches 1 as *a* increases.

The proportional overestimation error *ε* is given by

(19) $$\epsilon =\frac{{\mathrm{FWHM}}_{\text{gauss}}}{\mathrm{FWHM}}=\frac{2\sqrt{a}\sqrt{2log2}}{(a-1)\left[W_{0}\left(-\frac{2^{-1/(a-1)}}{e}\right)-W_{-1}\left(-\frac{2^{-1/(a-1)}}{e}\right)\right]}$$

Note that in Eq. (19), proportional error does not depend on the scale parameter *b*.

Table 1 shows proportional overestimation errors of the Gaussian approximation for different values of the shape parameter.

### 2.4. Octave bandwidth

The octave bandwidth is another frequently used characterization of a function’s width, that is closely related to the bandwidth (or FWHM) of a function (Iyer and Burge, 2019). The general form of octave bandwidth is given by

(20) $${BW}_{oct}={log}_{2}\left(\frac{H}{L}\right)$$

where *H* and *L* denote the high and low values that span the FWHM, respectively. Given expressions for the inverse of the probability density function (see. Eq. (9)) and the mode, obtaining an expression for the octave bandwidth is easy.

Here, we provide the expression for the octave bandwidth of gamma-distribution-shaped functions. As mentioned previously, when deriving the FWyM and FWHM, we found that *H* corresponds to the *k* = −1 branch and that *L* corresponds to the *k* = 0 branch of the Lambert W function

(21) $$H=-(a-1)bW_{-1}\left(-\frac{2^{-1/(a-1)}}{e}\right),\;L=-(a-1)bW_{0}\left(-\frac{2^{-1/(a-1)}}{e}\right)$$

Substituting Eq. (21) into Eq.(20) and simplifying yields

(22) $${\mathrm{BW}}_{\mathrm{oct}}=\log_{2}\left(\frac{W_{-1}\left(-2^{-1/(a-1)}/e\right)}{W_{0}\left(-2^{-1/(a-1)}/e\right)}\right)$$

Eq. (22) has appeared previously in the literature, but has not been described as the octave bandwidth of a gamma-distribution-shaped function. It has been reported as the frequency-domain octave bandwidth of a Cauchy filter (Boukerroui et al., 2004). The Cauchy filter in Fourier domain takes the form of a gamma distribution, which may be why this equation appears without any explicit mention of the gamma distribution.

Eq. (22) specifies the octave bandwidth, the log-base-2 ratio of the high and low values (see Eq. (21)) that span the full width at half maximum of the function. As is the case with the full width, the octave bandwidth can be generalized to an arbitrary proportion *y* of the maximum value of the function. For gamma-shaped functions, the generalized octave bandwidth is given by

(23) $${\mathrm{BW}}_{\mathrm{oct}}(y)=\log_{2}\left(\frac{W_{-1}\left(-y^{-1/(a-1)}/e\right)}{W_{0}\left(-y^{-1/(a-1)}/e\right)}\right)$$

### 2.5. Future work

Future work may consider investigating the bandwidth of the generalized gamma distribution, a super-family distribution which contains the gamma probability density as a special case (Stacy, 1962). Deriving the bandwidth of generalized gamma distributions may not be possible outside the special case of the gamma distribution. If so, a proof to this effect could be valuable.

### 2.6. MATLAB function

A MATLAB function is provided which computes the FWyM (and, hence, FWHM) of a gamma distribution using the formulae derived in this paper (see https://www.mathworks.com/matlabcentral/fileexchange/181960-gamma-distribution-bandwidth). The function takes in four arguments: the shape (*a* > 1) and scale (*b* > 0) parameters, the y-max (0 < *y* < 1) parameter, and an optional plotting parameter. The function returns three values: the FWyM, the low FWyM component, and the high FWyM component. The function depends on MATLAB’s Symbolic Math Toolbox, which includes an implementation of the Lambert W function.

## 3. Discussion and conclusion

Many kinds of data and signals are well characterized by functions taking their shape from gamma distribution PDFs. The full width at half-maximum (FWHM) is a common measure of the bandwidth of unimodal functions. We have derived a general expression for the FWHM of gamma-distribution-shaped functions for all cases in which the FWHM is well-defined (*a* > 1, *b* > 0). We derived the inverse of the gamma probability density function and used it to derive an expression for the FWyM–the full-width at an arbitrary *y*-proportion of the maximum. The expression for the FWHM follows trivially.

The expressions provided here should make simpler the accurate reporting of FWHM of gamma-distribution-shaped functions. We have provided a MATLAB function to aid such reporting.

## Supplementary Material

1

2

## Figures and Tables

**Table 1:** Gaussian approximation errors for various values of the shape parameter *a*. The approximation error does not depend on scale parameter *b*.

Shape parameter *a*	Proportional error *ε*	Gamma FWHM	Approximation
1.25	1.9328	1.3622	2.6328
2.5	1.2585	2.9586	3.7233
5	1.1073	4.7551	5.2655
10	1.0496	7.0947	7.4466
25	1.019	11.5547	11.7741
50	1.0094	16.4967	16.6511
100	1.0046	23.4393	23.5482

## Data Availability

No data was used for the research described in the article.

## Footnotes

1 The definition of FWHM is consistent across fields. But what is reported as the FWHM can vary with the specific application. As one example, when analyzing periodic functions (or signals) that have been windowed by modulating envelopes (e.g. wavelets), it is common to report the FWHM of the modulating envelope rather than of the modulated function (or signal) itself. This is common for applications in vision science, signal processing, quantum- and opto-electronics, among many other fields (Lu et al., 2005; Whitney and Bressler, 2007).

2 There are infinitely many branches of the Lambert W function. Each is indexed by a unique integer *k*, and each inverts an expression of the form *u* exp(*u*). When considering real-valued inputs and outputs, only the*k* = 0 and *k* = −1 branches are relevant. All other branches return complex numbers, and are hence not relevant for the present purposes.

3 Eq. (9) resembles the quantile function of the Gamma(2, *b*) distribution (see supplement), in that they are both written in terms of the *k* = −1 branch of the Lambert W function. The median can be computed from the quantile function. Studies of the gamma distribution’s median have made important contributions in a number of areas of mathematics.

## Appendix A. Supplementary data

Supplementary material related to this article can be found online at https://doi.org/10.1016/j.spl.2026.110707.
