# Supplementary material for: Bandwidth of gamma-distribution-shaped functions via Lambert W function
Source: Stat Probab Lett. Author manuscript; Available in PMC 2026 Jun 11. (PMC13246144; doi:10.1016/j.spl.2026.110707)
Supplement: 2 [file NIHMS2176872-supplement-2.pdf]

Supplement:
Bandwidth of Gamma-Distribution-Shaped Functions via Lambert W Function

Anthony LoPrete[a], Johannes Burge[a,b,c]

[a] *Bioengineering Graduate Group,* [b] *Neuroscience Graduate Group,* [c] *Department of Psychology, University of Pennsylvania, Philadelphia, Pennsylvania, USA*

---

1. Special-Case Equivalence of Quantile and Inverse Density Functions

The gamma distribution's median, $\upsilon(a, b)$, is the value at which the gamma distribution's cumulative distribution function (CDF, notated $F(x)$) equals one half

$$F\left(\nu(a, b)\right) = \frac{1}{2} \quad \text{where} \quad F(x) = \int_0^x \frac{1}{\Gamma(a)b^a} x^{a-1} e^{-x/b} dx \tag{S1}$$

Neither the median nor the CDF of the gamma distribution have known closed-form expressions in terms of elementary functions for all values of the shape parameter.[1] It is useful to have the important distributional characteristics (median, mean, variance, etc.) for probability distributions well-understood if not defined exactly, rendering the gamma distribution's median an active area of research. The median is known to be bounded by $a - 1/3 < \upsilon(a, 1)/b < a$, a result that can be shown analytically [1]. These bounds can be tightened under certain circumstances using numerical methods [2].

The equation of the inverse of the gamma distribution PDF (main document, Eq. 9) has an interesting relationship to the median of the gamma distribution in the special case in which $a = 2$. For this special case, the median does have a known closed-form expression. In what follows, we will show the nature of this relationship.

Before proceeding, we also note that studies of the gamma distribution's median hold special interest, in part because of the median's connection to an equation of Srinivasa Ramanujan. Namely, the Laurent series expansion for the median of the gamma distribution can be derived from Ramanujan's theta function [3, 4]. The Ramanujan theta function has applications to many important problems in discrete mathematics and computer science, including hashing, caching, the birthday paradox, and resource contention [5]. So progress on expressions for the median may benefit other areas of mathematics and computer science.

---

[1] The scale parameter's only influence on the gamma distribution's median is a scaling of the form $\upsilon(a, b) = b * \upsilon(a, 1)$.

We begin by extending consideration of the distribution's median to the more general quantile function. The quantile function of a random variable, denoted $F^{-1}(p)$, is defined as the inverse of the random variable's CDF. For gamma distributions, observing that $F(x)$ is strictly monotonic, we can write

$$F^{-1}(p) = x \quad \text{such that} \quad F_X(x) = p \tag{S2}$$

The problem of finding the gamma distribution's median can now be reduced to deriving an expression for $F^{-1}(p)$ and then evaluating $F^{-1}(1/2)$. As with the CDF, the gamma distribution's quantile function has no known closed-form expression for all values of the shape parameter, a. For the special case of Gamma$(2, b)$ distribution, however, the CDF can be written as

$$F(x; a = 2, b) = 1 - \frac{(b + x)e^{-x/b}}{b} \tag{S3}$$

Eq. S3 contains a product exponential. The quantile function of the Gamma$(2, b)$ can therefore be written in terms of the Lambert W function (see main text introduction) [6]:

$$F^{-1}(p; a = 2, b) = -b\left(1 + W_{-1}\left(\frac{p-1}{e}\right)\right) \tag{S4}$$

Eq. S4 bears a resemblance to Eq. 9 of the main document. Substituting $a = 2$ and $k = -1$ into Eq. 9 of the main text yields

$$f_k^{-1}(p; a = 2, b)\big|_{k=-1} = -bW_{-1}\left(-b * p\right) \tag{S5}$$

Equations S4 and S5 are both linear transformations of branch $k = -1$ Lambert W Functions. Resultantly, simple transformations of Equations S4 and S5 can render an equality relationship.

Recall that $f^{-1}(p)$ is multivalued for real inputs and real outputs. First, we scale the input probability by a factor of $1/(e * b)$. To isolate the greater of the two values outputted by $f^{-1}(p)$ (i.e. the value corresponding to the $k = -1$ branch) we apply the max function to $f^{-1}(p)$. Lastly, we scale the max function output by $1/b$. Applying these transformations to $f^{-1}(p; a = 2, b)$ gives

$$\begin{aligned} F^{-1}(1-p; a = 2, b)/b + 1 &= -b\left(1 + W_{-1}\left(\frac{1-p-1}{e}\right)\right)/b + 1 \\ &= -W_{-1}\left(\frac{-p}{e}\right) \end{aligned} \tag{S6}$$

The requisite transformation of the gamma distribution quantile function for $a = 2$ requires that one

substitute $1-p$ for p, scale the output of the quantile function by $1/b$, and then add 1, which yields

$$\begin{aligned} F^{-1}(1-p;a=2,b)/b+1 &= -b\left(1+W_{-1}\left(\frac{1-p-1}{e}\right)\right)/b+1 \\ &= -W_{-1}\left(\frac{-p}{e}\right) \end{aligned} \tag{S7}$$

The right-hand sides of Equations S6 and S7 are the same. Hence,

$$\max\left[f_k^{-1}(p/(e*b);a=2,b)\right]/b = F^{-1}(1-p;a=2,b)/b+1 \tag{S8}$$

Thus, in the special case in which the shape parameter $a=2$, we have established that for all values of p—and hence for the value (i.e., $p=1/2$) that corresponds to the median—a simple algebraic transform of the inverse density can be set equal to a simple algebraic transform of the quantile function.

References

[1] J. Chen, H. Rubin, Bounds for the difference between median and mean of gamma and poisson distributions, Statistics & Probability Letters 4 (6) (1986) 281–283. doi:https://doi.org/10.1016/0167-7152(86)90044-1.

[2] R. F. Lyon, On closed-form tight bounds and approximations for the median of a gamma distribution, PLOS One 16 (5) (2021) e0251626. doi:https://doi.org/10.1371/journal.pone.0251626.

[3] K. P. Choi, On the medians of gamma distributions and an equation of ramanujan, Proceedings of the American Mathematical Society 121 (1) (1994) 245–251. doi:https://doi.org/10.1090/S0002-9939-1994-1195477-8.

[4] S. Ramanujan, Question 294, Journal of the Indian Mathematical Society 3 (1911) 128.

[5] P. Flajolet, P. J. Grabner, P. Kirschenhofer, H. Prodinger, On ramanujan's q-function, Journal of Computational and Applied Mathematics 58 (1) (1995) 103–116. doi:https://doi.org/10.1016/0377-0427(93)E0258-N.

[6] F. Jiménez, P. Jódra, On the computer generation of the erlang and negative binomial distributions with shape parameter equal to two, Mathematics and Computers in Simulation 79 (5) (2009) 1636–1640. doi:https://doi.org/10.1016/j.matcom.2008.07.010.
